# Applying the win ratio method in clinical trials of orphan drugs: an analysis of data from the COMET trial of avalglucosidase alfa in patients with late-onset Pompe disease

**DOI:** 10.1186/s13023-023-02974-1

**Published:** 2024-01-12

**Authors:** Matthias Boentert, Kenneth I. Berger, Jordi Díaz-Manera, Mazen M. Dimachkie, Alaa Hamed, Lionel Riou França, Nathan Thibault, Pragya Shukla, Jack Ishak, J. Jaime Caro

**Affiliations:** 1 Department of Neurology and Institute of Translational Neurology, Münster University Hospital, Münster, Germany; 2 Division of Pulmonary, Critical Care and Sleep Medicine, New York University Grossman School of Medicine, New York, NY USA; 3 John Walton Muscular Dystrophy Research Centre, Newcastle University Centre for Life, Newcastle Upon Tyne, UK; 4 Department of Neurology, University of Kansas Medical Center, Kansas City, KS USA; 5 Sanofi, Cambridge, MA USA; 6 Aixial, Boulogne-Billancourt, France; 7 Evidera, Montreal, Canada; 8 Evidera, Boston, MA USA; 9 McGill University, Montreal, QC Canada; 10 London School of Economics, London, UK; 11 Evidera, 500 Totten Pond Rd, Waltham, MA 02451 USA

**Keywords:** Late-onset Pompe disease, Clinical trial, Win ratio, Enzyme replacement therapy, Avalglucosidase alfa

## Abstract

**Background:**

Clinical trials for rare diseases often include multiple endpoints that capture the effects of treatment on different disease domains. In many rare diseases, the primary endpoint is not standardized across trials. The win ratio approach was designed to analyze multiple endpoints of interest in clinical trials and has mostly been applied in cardiovascular trials. Here, we applied the win ratio approach to data from COMET, a phase 3 trial in late-onset Pompe disease, to illustrate how this approach can be used to analyze multiple endpoints in the orphan drug context.

**Methods:**

All possible participant pairings from both arms of COMET were compared sequentially on changes at week 49 in upright forced vital capacity (FVC) % predicted and six-minute walk test (6MWT). Each participant’s response for the two endpoints was first classified as a meaningful improvement, no meaningful change, or a meaningful decline using thresholds based on published minimal clinically important differences (FVC ± 4% predicted, 6MWT ± 39 m). Each comparison assessed whether the outcome with avalglucosidase alfa (AVA) was better than (win), worse than (loss), or equivalent to (tie) the outcome with alglucosidase alfa (ALG). If tied on FVC, 6MWT was compared. In this approach, the treatment effect is the ratio of wins to losses (“win ratio”), with ties excluded.

**Results:**

In the 2499 possible pairings (51 receiving AVA × 49 receiving ALG), the win ratio was 2.37 (95% confidence interval [CI], 1.30–4.29, *p* = 0.005) when FVC was compared before 6MWT. When the order was reversed, the win ratio was 2.02 (95% CI, 1.13–3.62, *p* = 0.018).

**Conclusion:**

The win ratio approach can be used in clinical trials of rare diseases to provide meaningful insight on treatment benefits from multiple endpoints and across disease domains.

**Supplementary Information:**

The online version contains supplementary material available at 10.1186/s13023-023-02974-1.

## Background

As rare diseases can affect several organ systems, multiple endpoints may be of interest in clinical trials. Often, primary outcomes are not standardized across trials. Thus, an analytic approach that recognizes the multiplicity of comparisons is needed. One option is to specify a hierarchical sequence for analyzing the individual endpoints [1], but if the effect on one endpoint in the sequence is not significant, no confirmatory claims can be made for all remaining endpoints in the hierarchy even if they would be significant on their own. Thus, important benefits may be missed. An alternative to a hierarchical sequence is to employ a composite endpoint that encompasses all outcomes of interest [1]. A potential limitation is that this considers only the first event to occur. Further, a composite endpoint does not account for the relative importance of the components (e.g., non-fatal events vs. death).

In 2012, Pocock et al. proposed the win ratio approach [2] to improve analyses of composite endpoints in randomized controlled clinical trials. The approach involves determining whether a participant on an experimental treatment did better (a “win”), worse (a “loss”), or about the same (a “tie”) as a participant on the control treatment across the series of endpoints, ordered by importance. In case of a tie on the most important endpoint, the next most important endpoint is compared. The wins and losses are tallied across all possible pairs of participants, and then the ratio of wins to losses is computed, reflecting the relative treatment effect of the investigational treatment versus its comparator. By considering multiple endpoints jointly, the win ratio approach produces a comprehensive assessment of the benefits of a treatment while enhancing statistical power. Further, it allows endpoints across different disease domains to be compared and can include different types of outcomes such as time-to-event, dichotomous, and continuous measures. Win ratios have been mostly used for analysis of data from cardiovascular clinical trials [3], and more recently in oncology [4] and COVID-19 trials [5–8], but this approach may also be helpful in trials with small sample sizes that must consider multiple clinical manifestations.

In this study, we aimed to illustrate how the win ratio approach can be used to analyze composite endpoints in the orphan disease context. To do this, we applied the win ratio approach to data from a trial (COMET; NCT02782741) in late-onset Pompe disease (LOPD; ORPHA:420429), a rare, progressive autosomal recessive glycogen storage disorder caused by bi-allelic pathogenic variants in the *GAA* gene encoding the lysosomal alpha-glucosidase [9, 10]. LOPD is a phenotype of Pompe disease (ORPHA:365) that generally presents after the first year of life and progressively reduces mobility and respiratory muscle function [11]. Many patients eventually need to use a wheelchair, home ventilatory support, or both [12]. Although a curative treatment is not available, enzyme replacement therapy using alglucosidase alfa (ALG) [13] has been shown to improve survival and maintain mobility and respiratory function in patients with LOPD [14].

COMET was a phase 3 randomized clinical trial [15] that compared avalglucosidase alfa (AVA) with ALG [13, 14, 16] for the treatment of LOPD [17]. The primary endpoint in COMET was improvement in respiratory function as measured by the change from baseline at week 49 in upright forced vital capacity (FVC) % predicted. A key secondary endpoint was improvement in mobility as measured by the six-minute walk test (6MWT). COMET employed a sequential statistical approach [15], wherein non-inferiority of AVA was assessed first, and if non-inferiority was demonstrated, superiority was tested sequentially on the primary and secondary endpoints. In this study, the win ratio approach was applied as an alternative analysis to explore the use of this method in a rare disease.

## Methods

### Study design

Data from COMET were reanalyzed with the win ratio approach to assess the overall effect of treatment on the primary endpoint (FVC % predicted) and the key secondary endpoint (6MWT). Details of the COMET trial design were published previously [15]. Briefly, this was a phase 3, randomized, double-blind trial that took place at 55 sites across 20 countries. Eligibility for COMET required a diagnosis of LOPD confirmed by acid alpha-glucosidase enzyme deficiency, two confirmed pathogenic *GAA* gene variants, or both; age ≥ 3 years; no previous treatment with Pompe-specific enzyme replacement therapy; and the ability to perform repeated FVC measurements of 30–85% predicted and walk ≥ 40 m without stopping or assistance. Patients were excluded if they required invasive ventilation or were wheelchair-dependent. COMET consisted of a 49-week double-blind primary analysis treatment period followed by an open-label extended treatment period. During the double-blind treatment period, participants were randomly allocated (1:1) to intravenous AVA or ALG, both administered at a dose of 20 mg/kg. FVC % predicted was calculated as the ratio of the actual to the predicted upright forced vital capacity for each participant based on their sex, race, age, and height. 6MWT distance was defined as the distance walked, in meters, over a span of 6 min.

### Statistical analysis

The win ratio analysis was based on the primary analysis population in COMET: the modified intent-to-treat population, which included all randomized participants who received at least one partial or full infusion of study drug. Prior to initiating the win ratio analysis, the endpoints were defined based on the COMET protocol: the change from baseline at week 49 in FVC % predicted was first in sequence and the change from baseline at week 49 in 6MWT distance was second.

Wins and losses were determined based on whether participants achieved meaningful improvement or worsening, defined as changes exceeding defined thresholds for each endpoint. The thresholds for meaningful differences (Table 1) were set prior to conducting the analysis using the midpoints of published minimal clinically important differences for FVC (2–6% predicted) and 6MWT (24–54 m) for chronic respiratory diseases, as applied in previous studies in Pompe disease [18].

**Table 1:** Thresholds for defining response levels

Change from baseline at week 49 in	Meaningful improvement	No meaningful improvement or decline	Meaningful decline
FVC, % predicted	Increase ≥ 4%	Increase or decrease < 4%	Decrease ≥ 4%
6MWT	Increase ≥ 39 m	Increase or decrease < 39 m	Decrease ≥ 39 m

*6MWT* 6-min walk test, *CI* confidence interval, *FVC* forced vital capacity

**Fig. 1:**
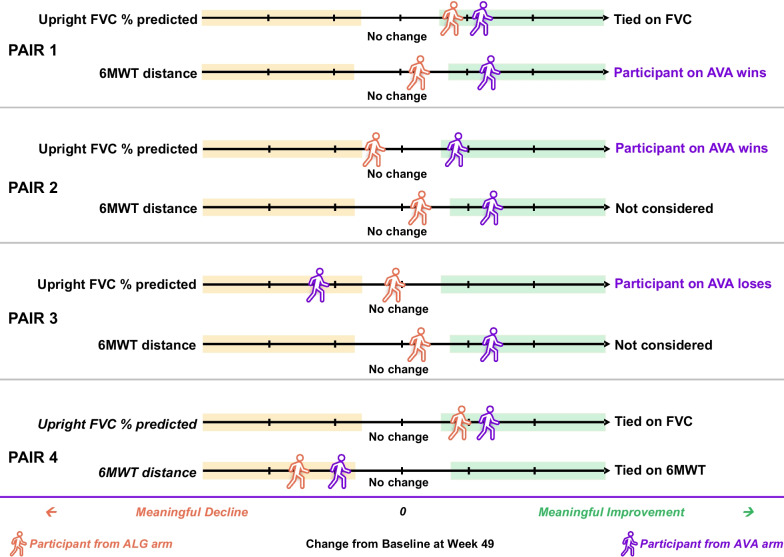
Conceptual diagram illustrating comparison of pairs of participants in the win ratio analysis. As a first step in win ratio calculation, each participant’s response for the two endpoints was classified as a meaningful improvement, no meaningful change, or a meaningful decline based on the midpoints of published minimal clinically important differences for FVC and 6MWT for chronic respiratory diseases as applied in previous studies in Pompe disease (see Table 1). In this conceptual diagram, the comparison of FVC % predicted is tied for the first pair of participants, as both participants had a meaningful improvement; the comparison therefore moves to the 6MWT, which results in a win for AVA because the participant from this group had meaningful improvement, while the participant on ALG had no meaningful change. In the second pair, the participant on AVA had a meaningful improvement in FVC % predicted while the participant on ALG had no meaningful change on this endpoint; this results in a win for AVA, and 6MWT is not considered. The third pair ends in a loss for AVA because the other participant in the pair had a better response on FVC % predicted. The fourth pair ends in a tie because responses were in the same category for both FVC % predicted and 6MWT. Abbreviations: 6MWT, 6-min walk test; ALG, alglucosidase alfa; AVA, avalglucosidase alfa; FVC, forced vital capacity

As a first step in the win ratio calculation, each participant’s response for the two endpoints was classified as a meaningful improvement, no meaningful change, or a meaningful decline. Next, all participants receiving ALG were paired with all in the AVA arm, and the response was compared for each pair. A win occurred when the AVA participant had a better response than the ALG participant; a loss occurred if the response was worse for the AVA participant; and a tie occurred when the response was the same for both participants. Figure 1 illustrates some hypothetical scenarios for how pairs of participants could be compared.

The win ratio was calculated as total wins divided by total losses across all pairs. Multiple imputation was used to assess the impact of missing data [19]. In the analysis based on observed data, if one of the pair died or dropped out of the study prior to week 49 the comparison was considered a tie. For multiple imputation, five imputed data sets were created for the change from baseline at week 49 in participants who were alive at week 49 or who were lost to follow-up prior to week 49. Participants who died during the trial were excluded from imputation. Imputations were based on age at baseline, sex, treatment group, and baseline and longitudinal values of the outcome variables (including at weeks 13, 25, and 37). Win ratios were calculated for each imputed data set and were pooled to obtain an overall result. In another sensitivity analysis, the win ratio was recalculated without multiple imputation using the reverse order of comparisons (i.e., 6MWT first, followed by FVC). Win ratios were also calculated within subgroups based on sex and baseline FVC % predicted (< 55% and ≥ 55%), ordering the endpoints the same as in the primary analysis. Further details of the win ratio calculation are provided in Additional File 1.

## Results

### Study sample

The modified intent-to-treat COMET population included 100 participants, 51 randomized to AVA and 49 to ALG [15]. Baseline demographics and clinical characteristics were similar across the two groups (Table 2).

**Table 2:** Baseline demographic and clinical characteristics in the modified intent-to-treat population of COMET

Characteristic	Overall (N = 100)	AVA (N = 51)	ALG (N = 49)
Mean age (SD), years	48 (14)	46 (14)	50 (14)
Male, n (%)	52 (52)	27 (53)	25 (51)
Race, n (%)			
Asian	3 (3)	3 (6)	0 (0)
Black or African American	3 (3)	1 (2)	2 (4)
White	94 (94)	47 (92)	47 (96)
Mean age at symptom onset (SD), years	35 (16)^a^	33 (17)^a^	38 (16)
Mean age at diagnosis (SD), years	46 (15)	45 (15)	48 (15)
Mean FVC (SD), % predicted	62.1 (13.4)	62.6 (14.4)	61.6 (12.4)
Mean 6MWT (SD), m	388.9 (113.5)	399.3 (110.9)	378.1 (116.2)

The modified intent-to-treat population included all randomized participants who received at least one partial or full infusion of study drug. Full baseline demographics and clinical characteristics were published in Diaz-Manera et al. 2021 [15]

*6MWT* six-minute walk test, *ALG* alglucosidase alfa, *AVA* avalglucosidase alfa, *FVC* forced vital capacity, *SD* standard deviation

^a^Overall based on n = 99; AVA based on n = 50

All 51 participants randomized to AVA and 44 of the 49 randomized to ALG completed the 49-week primary analysis period. One participant randomized to ALG withdrew consent and four permanently discontinued due to adverse events. Two participants in the AVA group were missing values for change from baseline in FVC % predicted, compared to six in the ALG group. Similarly, three in the AVA group were missing change from baseline in 6MWT, compared to six in the ALG group.

### Win ratio analysis

Of the 2499 (51 × 49) pairings of participants, 923 resulted in a win for AVA on FVC, 417 resulted in a loss, and 1159 resulted in a tie (Table 3). These 1159 ties were then compared on 6MWT, yielding 314 wins for AVA, 106 losses, and 739 ties. Across the two endpoints, there were 1237 wins for AVA and 523 losses, resulting in a win ratio of 2.37 (95% confidence interval [CI], 1.30–4.29; *p* = 0.005). The win ratio was similar (2.10; 95% CI: 1.18–3.73; *p* = 0.012) when missing endpoint values were assigned with multiple imputation (Additional File 2). When the order of comparisons was reversed (i.e., 6MWT first, followed by FVC), the win ratio was 2.02 (95% CI: 1.13–3.62; *p* = 0.018) (Table 3).

**Table 3:** Win ratio analysis comparing pairs of patients on clinically meaningful improvement or decline in the two measures in the order indicated

Measure	Order of comparison of endpoints	
	FVC % Predicted 6MWT	6MWT FVC % Predicted
Comparison based on the first endpoint in the sequence		
Wins	923	786
Losses	417	330
Ties	1159	1383
Comparison based on second endpoint in the sequence		
Wins	314	391
Losses	106	253
Ties	739	739
Totals		
Wins	1237	1177
Losses	523	583
Ties	739	739
Win ratio (95% CI)	2.37 (1.30, 4.29)	2.02 (1.13, 3.62)
*p*-value	0.005	0.018

Win ratio is the total wins divided by the total losses

*6MWT* six-minute walk test, *CI* confidence interval, *FVC* forced vital capacity

Win ratios calculated in subgroups defined by sex and baseline FVC % predicted (< 55% and ≥ 55%) were 2.03 (95% CI: 0.92–4.48) in men and 2.93 (95% CI: 1.19–7.22) in women; 1.21 (95% CI, 0.48–3.05) when the baseline FVC % predicted was < 55% and 3.75 (95% CI, 1.65–8.53) when ≥ 55% (Table 4).

**Table 4:** Win ratio analysis in subgroups defined by sex and baseline FVC

Subgroup	N		Win ratio (95% CI)	*p*-value
	AVA	ALG		
Sex				
Male	27	25	2.03 (0.92–4.48)	0.078
Female	24	24	2.93 (1.19–7.22)	0.020
Baseline FVC % predicted				
≥ 55%	35	30	3.75 (1.65–8.53)	0.002
< 55%	16	19	1.21 (0.48–3.05)	0.685

*ALG* alglucosidase alfa, *AVA* avalglucosidase alfa, *CI* confidence interval, *FVC* forced vital capacity

## Discussion

This re-analysis of outcome data from COMET illustrates the use and advantages of the win ratio analytic approach. By considering multiple endpoints jointly, the win ratio approach produces a more comprehensive assessment of the benefits of a treatment. It combines evaluation of several endpoints into a single analysis while avoiding double-counting of effects. This is achieved by comparing outcomes sequentially and only analyzing lower-ranking endpoints among participants who are tied on higher-ranking endpoints. By jointly considering different types of outcomes, the win ratio approach can provide increased statistical power to detect and quantify a treatment effect. This can be important for clinical trials in diseases with clinically heterogeneous manifestation, and especially for rare diseases, where sample sizes are typically small [3]. Another advantage of the win ratio approach is that it can consider response categories, which makes it less susceptible to outliers than parametric analysis of continuous endpoints.

The win ratio offers a different way of assessing treatment benefit than the sequential statistical approach used in the original COMET analysis [15] and can thus provide additional insights. The original analyses were based on comparing mean changes in outcomes using a sequential testing approach involving first demonstrating non-inferiority on the primary endpoint (FVC % predicted) and then evaluating each outcome separately for superiority, with testing continuing as long as statistical significance criteria were met. Thus, when superiority was not met on FVC % predicted, testing stopped and superiority on 6MWT could not be assessed. The win ratio maintains the sequential assessment of outcomes but adds the ability to simultaneously assess treatment responses in two or more disease domains as a single measure expressing how likely a patient is to benefit from the treatment.

It is important to reconcile differences between results in this post-hoc win ratio analysis and those obtained in the original COMET analysis. The pre-planned COMET analysis provided statistical support for non-inferiority of AVA, whereas the post-hoc win ratio analysis suggests that a patient receiving AVA is more than twice as likely to benefit than one receiving ALG. This apparent discrepancy arises from the specific properties of each of the statistical approaches and requires translation into patient care. This win ratio analysis provides a rationale for combining new insights with existing evidence from the COMET study and may lead clinical decision-makers to prefer AVA over ALG when initiating enzyme replacement therapy.

Another aspect that differs between the two analytic approaches is that the original analysis measures the treatment effect via the mean extent of change from baseline whereas the win ratio approach compares clinically meaningful improvement or decline. Making direct comparisons between the magnitudes of change (i.e., comparing absolute change values) would have led to decisions based on the first endpoint only and precluded ties, except in rare cases of numerically identical changes. This would have defeated the purpose of jointly assessing endpoints. Further, comparing exact magnitudes of change would have led to some wins and losses based on small, clinically insignificant differences, which would have masked meaningful effects of treatment.

The thresholds for meaningful improvement and decline used in this study were selected before any analyses were conducted and were based on published minimal clinically important differences for FVC and 6MWT that have been applied in other studies of LOPD [18]. An alternative approach, not used in the current analysis, would have been to determine the thresholds based on a minimum difference between the observed changes in FVC and 6MWT. In other words, a win would require exceeding a minimum difference in the change from baseline between the participants in each pairing. This was considered to be inconsistent with how patients would be evaluated clinically.

In a win ratio analysis, the order in which endpoints are compared is meant to reflect their clinical importance [2]. For clinical trials in which the endpoints have a clear order—for example, death followed by hospitalization in cardiovascular trials [20]—selecting the order of comparisons is straightforward. Whether FVC or 6MWT should be compared first is debatable. In fact, both have been used as primary endpoints in LOPD clinical trials [21–24]. We therefore retained the order specified in the COMET trial protocol as the main analysis and tested the reverse order in a sensitivity analysis. In both cases, participants receiving AVA were over twice as likely to have better outcomes than those receiving ALG, indicating that the order of comparison was not an important factor in the COMET analyses.

It is important to emphasize that the win ratio analysis was conducted post hoc. Post hoc approaches can be subject to false discovery and bias [25]; a main concern is that only re-analyses yielding favorable results may be reported. To help limit this concern, we selected the first two endpoints from the COMET study (FVC and 6MWT) and maintained their pre-specified hierarchy. In addition, the thresholds used for determining wins and losses and the methods for handling missing data (i.e., analyzing data as observed and with multiple imputation) were defined before any analyses were conducted.

A further limitation of this study was that some data were missing. This could be a concern for a small study like COMET, which included 100 participants. However, multiple imputation did not indicate bias as a result of missing data.

## Conclusions

This reanalysis of outcome data from the COMET trial highlights the win ratio as an analytic approach that can provide meaningful insight on treatment benefits across various disease domains in clinical trials of rare diseases where multiple endpoints are evaluated.

### Supplementary Information

**Additional file1** Calculation of the win ratio. Further details of the win ratio calculation

**Additional file2** Win ratio analysis based on multiple imputation. Table showing results of win ratio analysis based on multiple imputation

## Data Availability

Qualified researchers may request access to patient-level data and related study documents. Patient-level data will be anonymized and study documents will be redacted to protect the privacy of trial participants. Further details on Sanofi’s data sharing criteria, eligible studies, and process for requesting access can be found at https://www.clinicalstudydatarequest.com.

## Abbreviations

6MWT: Six-minute walk test
ALG: Alglucosidase alfa
AVA: Avalglucosidase alfa
CI: Confidence interval
FVC: Forced vital capacity
LOPD: Late-onset Pompe disease
