# Supplementary material for: Applying the win ratio method in clinical trials of orphan drugs: an analysis of data from the COMET trial of avalglucosidase alfa in patients with late-onset Pompe disease
Source: Orphanet J Rare Dis. 2024 Jan 12;19:14. doi: 10.1186/s13023-023-02974-1 (PMC10785533; doi:10.1186/s13023-023-02974-1)
Supplement: Supplementary file 1 — Additional file1 Calculation of the win ratio. Further details of the win ratio calculation [file 13023_2023_2974_MOESM1_ESM.docx]

# Additional File 1

## Calculation of the win ratio

The win ratio is based on the Finkelstein-Schoenfeld test, and the general approach includes creating all possible pairs in the trial, i.e., every participant on the new treatment is compared with every participant on the control treatment. Then, within each pair one evaluates the component outcomes in descending order of importance until one of the pair shows a better outcome compared with the other. If the participant on the new treatment has the better outcome it is called a ‘win’, whereas if the control participant does better it is a ‘loss’. Otherwise, it is a ‘tie’.

Specifically, if N_T_ and N_C_ be the number of participants in the treatment and control groups, respectively, then there will be N_T_ X N_C_ paired comparisons. The win ratio (R_W_) is then calculated as:

$$R_{W}= \frac{N_{W}}{N_{L}}$$

where N_W_ and N_L_ are the total number of pairwise wins and losses for treatment arm respectively. The p-value of this win ratio is then determined based on the approach introduced by Finkelstein and Schoenfeld :

The total sample size is given by N= N_T_ + N_C_. Irrespective of treatment group we compare all possible pairs of participants i,j to determine whether participant i was the winner, the loser, or they tied and assign u_ij_= +1, -1 or 0 according to whether participant i was the winner, the loser or they tied, respectively. Then for participant i, we define $U_{i}=\sum_{i\neq j} u_{ij}$. U_i_ will be a positive integer if participant i wins more often than he loses as compared to all other participants. We then calculate the statistic

$$T=\sum_{i=1}^{N} U_{i}D_{i}$$

where D_i_ = 1 if participant i is on the treatment and D_i_ = 0 if participant i is on the control. Under the null hypothesis of no true difference between treatment and control, T has variance V where

$V=\frac{N_{T}N_{C}}{N(N-1)}\sum_{i=1}^{N} U_{i}^{2}$.

Assuming that T follows a normal distribution, we can derive the z-score implied by *T* and *V* ($z=\frac{T}{\sqrt{V}})$, and obtain the p-value for a two-sided test for T given by 2xP(Z > z), where Z follows a standard normal distribution.

An approximate 95% CI for $R_{W}$ can also be calculated based on the z-score by first deriving the standard error (SE) for log R_w_:

$$SE\left( logR_{w} \right)= \frac{logR_{w}}{z}$$

The 95% CI around log R_w_ is then calculated as $logR_{w}\pm(1.96\times SE\left( logR_{w} \right))$. The limits are then exponentiated to derive CI around R_w_.

## Pooling of results from analyses with multiple imputation

Imputation yielded $m = 5$ datasets and win ratio analyses described applied on each of these. This produced five estimates of the win $R_{W,i}$ along with z-scores, p-values and 95% CI as described in the previous section. A pooled estimate of the win ratio across replications was calculated as the average of win ratios across the imputed datasets:

$\bar{R_{W}}= \frac{\sum_{i=1}^{m} R_{Wi}}{m}$.

The total variance of the pooled estimate was obtained by applying Rubin’s rule to account for both within and between replication variances. Calculations were based on the variance of the $T$ statistic as follows. Let $T_{i}$ and $V_{i}^{w}$ denote the estimate and variance of $T$ computed in the ith imputed dataset. The total variance of T is then given by

$V^{T}=\frac{\sum_{i=1}^{m} V_{i}^{w}}{m}+(1+\frac{1}{m})\frac{\sum_{i=1}^{m} {(T_{i}-\bar{T})}^{2}}{m-1}$.

The z score for the pooled estimate is then calculated as

$$z^{p}=\frac{\sum_{i=1}^{m} T_{i}}{m}\div\sqrt{V^{T}}$$

and the associated p-value based on normal distribution (2 x P(Z > $z^{p}$), where Z follows a normal distribution. The SE implied by the z-score is calculated as ${SE}^{p}= \log\left( \bar{R_{W}} \right)\div z^{p}$ and used to compute the 95% CI for the pooled win ratio as

$\exp\left( \log\left( \bar{R_{W}} \right)\pm1.96*SE \right)$.
