# Supplementary material for: Applying the win ratio method in clinical trials of orphan drugs: an analysis of data from the COMET trial of avalglucosidase alfa in patients with late-onset Pompe disease
Source: Orphanet J Rare Dis. 2024 Jan 12;19:14. doi: 10.1186/s13023-023-02974-1 (PMC10785533; doi:10.1186/s13023-023-02974-1)
Supplement: Supplementary file 2 — Additional file2 Win ratio analysis based on multiple imputation. Table showing results of win ratio analysis based on multiple imputation [file 13023_2023_2974_MOESM2_ESM.docx]

# Additional File 2

**Win ratio analysis based on multiple imputation**

|  | **Multiple imputation^a^** | | | | | | |
| --- | --- | --- | --- | --- | --- | --- | --- |
|  | **iterations** | | | | | | |
| **Outcome** | | **1** | **2** | **3** | **4** | **5** | **Pooled** |
| FVC % predicted | |  |  |  |  |  |  |
| Wins | | 1035 | 1036 | 1036 | 989 | 1010 | 1021 |
| Losses | | 489 | 498 | 498 | 535 | 526 | 509 |
| Ties | | 975 | 965 | 965 | 975 | 963 | 969 |
| 6MWT | |  |  |  |  |  |  |
| Wins | | 346 | 342 | 346 | 357 | 342 | 347 |
| Losses | | 150 | 150 | 122 | 153 | 148 | 145 |
| Ties | | 479 | 473 | 497 | 465 | 473 | 477 |
| Total | |  |  |  |  |  |  |
| Wins | | 1381 | 1378 | 1382 | 1346 | 1352 | 1368 |
| Losses | | 639 | 648 | 620 | 688 | 674 | 654 |
| Ties | | 479 | 473 | 497 | 465 | 473 | 477 |
| Win Ratio (95% CI) | | 2.16 (1.22, 3.82) | 2.13 (1.21, 3.75) | 2.23 (1.25, 3.96) | 1.96 (1.12, 3.42) | 2.01 (1.14, 3.52) | 2.10 (1.18, 3.73) |
| *p*-value | | 0.008 | 0.009 | 0.006 | 0.019 | 0.015 | 0.012 |

Win and loss counts represent the number of pairs ending in a win or loss for the participant on avalglucosidase alfa. Abbreviations: 6MWT, six-minute walking test; CI, confidence interval; FVC, forced vital capacity.

^a^ One participant treated with alglucosidase alfa died due to a serious adverse event of acute myocardial infarction considered unrelated to treatment and had no post-baseline measurements. For this participant no imputations were done.
